# *cnd-1*/NeuroD1 Functions with the Homeobox Gene *ceh-5*/Vax2 and Hox Gene *ceh-13*/labial To Specify Aspects of RME and DD Neuron Fate in *Caenorhabditis elegans*

**DOI:** 10.1534/g3.120.401515

**Published:** 2020-06-29

**Authors:** Wendy Aquino-Nunez, Zachery E. Mielko, Trae Dunn, Elise M. Santorella, Ciara Hosea, Lauren Leitner, Derrica McCalla, Claire Simms, Wendy M. Verola, Sharanya Vijaykumar, Martin L. Hudson

**Affiliations:** Department of Molecular and Cellular Biology, Kennesaw State University, Kennesaw, GA 30144

**Keywords:** NeuroD1, cell fate specification, Hox genes, homeobox genes, RNA-seq

## Abstract

Identifying the mechanisms behind neuronal fate specification are key to understanding normal neural development in addition to neurodevelopmental disorders such as autism and schizophrenia. *In vivo* cell fate specification is difficult to study in vertebrates. However, the nematode *Caenorhabditis elegans*, with its invariant cell lineage and simple nervous system of 302 neurons, is an ideal organism to explore the earliest stages of neural development. We used a comparative transcriptome approach to examine the role of *cnd-1*/NeuroD1 in *C. elegans* nervous system development and function. This basic helix-loop-helix transcription factor is deeply conserved across phyla and plays a crucial role in cell fate specification in both the vertebrate nervous system and pancreas. We find that *cnd-1* controls expression of *ceh-5*, a Vax2-like homeobox class transcription factor, in the RME head motorneurons and PVQ tail interneurons. We also show that *cnd-1* functions redundantly with the Hox gene *ceh-13*/labial in defining the fate of DD1 and DD2 embryonic ventral nerve cord motorneurons. These data highlight the utility of comparative transcriptomes for identifying transcription factor targets and understanding gene regulatory networks.

Accurate control of gene expression is fundamental for the development and function of the central nervous system (CNS). Defects in CNS gene expression underlie many neurodevelopmental disorders, indicating a critical need for further study (Basu *et al.* 2009; Schizophrenia Working Group of the Psychiatric Genomics Consortium 2014). Gene expression is controlled by combinations of transcription factors that work in conjunction with chromatin remodeling complexes to promote or inhibit RNA polymerase access to the genome (Clapier and Cairns 2009). According to Waddington’s model of cellular differentiation, cell fate progressively refines over multiple rounds of cell division, first to tissue-type progenitors, then exiting the cell cycle to take on a tissue-specific terminal fate (Waddington 1957). The transcription factors and chromatin remodeling complexes required for these narrowing rounds of fate specification are not well understood.

The basic Helix-Loop-Helix (bHLH) super family of proneural transcription factors includes Atonal, NeuroD, Neurogenin, and Achaete/Scute, and has broad roles in nervous system development (Baker and Brown 2018). This family of bHLH transcription factors function as either homodimers or heterodimers and bind to E-box sequences of the motif CANNTG. In *Drosophila*, the bHLH family acts in neural cell fate specification and neurogenesis, with different family members having roles in external sensory organ formation, chordotonal organ development, and others (Lee 1997).

Vertebrate *neurogenic differentiation 1* (NeuroD1) is a bHLH transcription factor that has a role in the transcriptional activation of proneural genes (Wang and Baker 2015). In addition, NeuroD1 is expressed abundantly in the brain after terminal fate specification, which suggests a secondary role in nervous system homeostasis and/or neural maturation and survival (Miyata *et al.* 1999). Ectopic expression of NeuroD1 in *Xenopus* embryos can convert non-neural ectodermal cells into fully differentiated neurons, indicating the potential role of NeuroD1 as a neural differentiator factor (Lee *et al.* 1995; Lee 1997). Humans bearing homozygous NeuroD1 mutations showed severe cerebral hypoplasia and developmental delay, in addition to defects in pancreatic β-cell maturation and islet formation, demonstrating the importance of this gene in nervous system and pancreatic development (Rubio-Cabezas *et al.* 2010). In the mouse, NeuroD1 is essential for the generation of granule cells in the hippocampus and the cerebellum (Miyata *et al.*1999; D’Amico *et al.* 2013). Despite extensive research on the role of NeuroD1 in cell fate specification and nervous system development (Seo *et al.* 2007; Pataskar *et al.* 2016), a comprehensive list of NeuroD1 targets has not been compiled and many questions on its role in neural development remain unanswered.

The nematode *Caenorhabditis elegans*, with its invariant cell linage and well-defined nervous system, is an excellent model to study cell lineage determination and terminal fate specification (Sulston and Horvitz 1977; Sulston *et al.* 1983). Once a neuroblast exits the cell cycle, it needs to extend growth cones and axons through the extra-cellular matrix, find its appropriate pre- and post-synaptic partners, assemble a synapse, gap junction, or neuromuscular junction, then package the various proteins required for synaptic transmission (Chisholm *et al.* 2016; Jin and Qi 2018). This leads to two major questions. First, what cascade of transcription factors is required to specify interim cell fates, prior to final specification of a neuron? Second, does a single transcription factor control the fate of a single neuron, or is terminal fate specified in a combinatorial manner, with multiple transcription factors controlling different aspects of the final cell fate? Extensive work has identified a battery of transcription factors known as “terminal selectors”, which are required for terminal fate specification in *C. elegans* neurons (Hobert 2016 and references therein). These transcription factors generally act in a combinatorial fashion to specify cell fates, although individual transcription factors may specify the fate of multiple cells that are unrelated by cell lineage, type, or circuit. Terminal selectors typically have autoregulatory properties, in that they positively regulate their own transcription to maintain neuronal identity throughout the life of an organism. In addition, they either directly or indirectly control the expression of “terminal effector” genes, which are required for that neuron’s post-mitotic function, for instance neurotransmitter biosynthesis, packaging, and release. Despite this depth of knowledge, the “proneural” transcription factors that act up-stream of terminal selector genes are not well described.

The *C. elegans* bHLH transcription factor *cnd-1* is orthologous to the human NeuroD1 gene and is one of the earliest proneural genes to be activated during *C. elegans* embryonic development (Hallam *et al.* 2000). However, the only reported defects seen in *cnd-1* loss-of-function mutants are a relatively mild back-coiler phenotype caused by misspecification of 2-3 dorsal D (DD) motorneurons required for inhibitory GABAergic neuromuscular innervation, in addition to axon guidance and synapse remodeling defects in the remaining D neurons (Hallam *et al.* 2000). To gain a better understanding of CND-1’s role during *C. elegans* neural development, we performed an RNA-seq assay comparing embryonic wild type and *cnd-1**(**ju29**)* mutant transcriptomes. We find that CND-1 positively regulates the expression of homeobox transcription factor *ceh-5*/Vax2 in the head RME and tail PVQ neurons. We also confirm that CND-1 is required for the generation of *cnd-1* expressing cells during ventral nerve cord fate specification. Finally, we show that *cnd-1* functions in parallel with the Hox gene *ceh-13*/labial to specify a subset of embryonic DD class ventral nerve cord motorneuron fates.

## Materials and Methods

### Strains and maintenance

*C. elegans* strains were grown on nematode growth medium plates (NGM Lite) at 20° according to Brenner (1974). Bristol N2 strain was used as wild type and all analyses were conducted at 20°. The following alleles were used in this study: LGIII *cnd-1**(**ju29**)*, *cnd-1**(**gk718**)* and *ceh-13**(**sw1**)/**qC1** [**dpy-19**(**e1259**) **glp-1**(**q339**)]*. Integrated transgenes used were *juIs76** [unc-25p*::*GFP + **lin-15**(+)]*, *kyIs39** [sra-6p*::*GFP + **lin-15**(+)]*, *lhIs5** [unc-25p*::*mCherry]*, *otIs356** [rab-3p(prom1)*::*2xNLS*::*TagRFP]*, *pkIs586** [gpa-9p*::*GFP + **dpy-20**(+)]*, and *stIs10055** [cnd-1p*::*his-24*::*mCherry + **unc-119**(**ed3**)]*. Extra-chromosomal arrays used in this study were *leEx2489** [ceh-5p*::*GFP + **unc-119**(+)]* and *dbEx724** [flp-6p*::*tax-2**(cDNA)*::*SL2*::*GFP + **lin-15**(+)]*. The *cnd-1**(**gk718**)* allele was identified by the *C. elegans* deletion mutant consortium (2012). All mutants were outcrossed at least twice prior to analysis. Table S1 shows details of strains generated during the course of this study including strain numbers and sources.

A *cnd-1**(**gk718**) **ceh-13**(**sw1**)/**qC1* line was built by crossing *gk718**/+* males into *sw1**/**qC1*, keeping lines that did not give rise to the *qC1* dumpy/sterile phenotype (genotype *gk718** +/ + **sw1**)*, selecting for *gk718* homozygous animals (uncoordinated phenotype) then screening for the embryonic lethal *sw1* phenotype (parent genotype *gk718* *sw1**/ **gk718** +)*. Two recombinants were identified from 200 *gk718* animals screened, consistent with the 1 map unit distance between *cnd-1* and *ceh-13* on LG III. These lines were rebalanced over the *qC1* chromosomal inversion prior to further characterization.

### RNA extraction

Embryos were isolated from gravid worms grown in liquid culture as described previously (Hudson *et al.* 2006). Total RNA was extracted using RiboZol (AMRESCO) and followed the vendor’s protocol except that embryos were frozen in liquid nitrogen prior to grinding. Embryonic tissue was added to 1 mL of RiboZol and 500 μL aliquoted into two 5 PRIME Phase Lock Gel Tubes. At the isopropanol stage, 3μL of 20ng/ml Glycogen was added to improve RNA pellet visualization. RNA quality control was assayed by measuring the A260/A280 ratio using a Thermo Scientific NanoDrop and via an Agilent Bioanalyzer. All samples used for RNA-Seq had an RNA integrity number (RIN) above 9.2. Triplicate *cnd-1*(*ju29*) and N2 wild type samples were sent to the University of Kansas Genome Sequencing Core for RNA-seq library construction (Illumina TruSeq v2), barcoded, pooled, and sequenced in a single lane on an Illumina HiSeq 2500 system (high output, single read 100bp sequencing).

### RNA-Seq expression analysis pipeline

Gene expression abundance were obtained using previous published RNA-seq analysis workflows. Briefly, read quality was determined using FastQC 0.11.5 (Andrews 2010). Reads were aligned back to the *C. elegans* reference genome (wbcel235) using Hisat2-2.1.0 (Kim *et al.* 2015). Samtools 1.5 (htslib 1.4.1) was used for file conversion and sorting (Li *et al.* 2009). Stringtie 1.3.3.b was used to assemble and quantify transcripts (Pertea *et al.* 2016). The script prepDE.py was used to generate gene and transcript count matrix files that were compatible with DESeq2 1.16.1 (R3.4.1), which was used to identify differentially expressed genes (Love *et al.* 2014). All programs were run locally.

### Identification of differential isoform usage

This was performed using DEXSeq (Anders *et al.* 2017). Raw RNA-seq reads were mapped with HiSat2 to the *C. elegans* annotated reference genome obtained from Ensembl. A python script provided by the DEXSeq package was used to combine all isoforms of a gene into one global schematic representation with marked intron/exon boundaries. The analysis was performed following the developers’ recommendations with the following inclusion “- s” for no strand specific reads.

### Characterization of the ken2 deletion allele

2.0X Taq RED Master Mix Kit (Apex Bioresearch Products) was used to amplify the 3′ end of the *ptrn-1* gene. Reverse primers at approximately 1kb intervals were used in combination with a

single forward primer within the *ptrn-1* coding region to amplify the *ptrn-1* 3′ untranslated region (UTR). Primer R9, located 10kb downstream of the F1 forward primer were the only pair which amplified *cnd-1**(**ju29**)* genomic DNA, and gave a 3kb amplicon. Sanger sequencing was used to confirm the *ken2* breakpoints, which corresponds to a 6908bp deletion/80bp insertion. Coordinates of the *ken2* breakpoints along with insertion and flanking sequences are; LG X

AGAGATACACATGTTTTTGGTGCTTTGTAGAAACCAGTACACGCGCATTTTCACTTACTTTTTTTATTTTTTTCCGTTTCTTTCTGTTTCTAATTTTGCAGATT (17,022,165)/

CTAATTTTGCAGATTCGGTGTTCTCCGAGGTTTTTTAAATCGGTGGGCAGGTGGAAATATTTTGTCATAGTTTTTCGAAG / (17,015,257)

TATCAGGTTGTCCCATAAGTTTTTGTACTATTTTTTTTTTTGAAAAAAATTTATTTCTCTCAAGCGACAAGTAGTACTATTCACACAAGTATTCACCATTAGTGT. Note that the 3′ breakpoint lies in a transposon sequence and is difficult to identify via BLAST search. The *ken2* deletion removes all of *F35B3.1*, *F35B3.10*, *F35B3.4*, and around 250bp of the *ptrn-1* 3′ untranslated region. Primer sequences used for PCR characterization of the *ken2* allele were as follows and show expected wild type amplicon size:

ptrn-1F1 (gtgaccaaatccaaccgtg); ptrn-1R1 (ttgcctcagtgcattttgg, 593bp); ptrn-1R2 (ctttaaggaaggcatgggatg, 1164bp); ptrn-1R4 (gtcaaggcatcaagtggttag, 2245bp); ptrn-1R5 (tttgaataatgcctctttaaagtgaatg, 3670bp); ptrn-1R6 (aggtaactttctgagcccac, 4717bp); ptrn-1R7 (gtggaacctgaagtgaataatgg, 6116bp); ptrn-1R8 (caaaaccgtctgccacg, 7119bp); ptrn-1R9 (gaagtagtagatcagccatatgc, 9896bp).

### Plasmid constructs

Plasmids were constructed using Gateway Technology (Invitrogen). Target gene promoters were PCR amplified from genomic DNA using Phusion polymerase (Thermo Scientific), gel purified, A-overhangs added by incubating with Taq RED Master Mix Kit (Apex Bioresearch Products), cloned into the pCR8/GW/TOPO vector (Thermo Scientific) following the manufacturer’s specifications then transformed into NEB 5-alpha Competent *E. coli* (New England BioLabs). Plasmid digestion was used to confirm the forward orientation of the amplicon. Promoters were recombined into the promoterless Gateway GFP expression vector pCZGY32 vector (a kind gift from Yishi Jin, UC San Diego) using Gateway LR Clonase II Enzyme Mix (Invitrogen) following the vendor’s instructions and transformed into NEB 5-alpha Competent *E. coli*. Plasmid digestion was used to confirm identity. Constructs were microinjected into the germlines of either wild type or *stIs10055**[ cnd-1p*::*his-24*::*mCherry **unc-119**(+)]* worms at 20ng/μL in conjunction with either *unc-122*::*GFP* (coelomocyte::GFP) or *ttx-3p*::*RFP* co-injection plasmids and pBlueScript II to a final concentration of 50ng/μL. F1 hermaphrodites that expressed the co-injection marker were single plated and screened to obtain stable lines.

### cDNA synthesis and RT-qPCR

Total RNA from mixed staged embryos was treated with DNAase (New England Biolabs) and cleaned using the RNA Clean &amp;amp;amp; Concentrator kit (Zymo Research). First-strand synthesis was done using the RevertAid First Strand cDNA Synthesis Kit (Thermo Scientific). *cdc-42* transcript levels were used to normalize for differences in input cDNA. Three or four biological samples, in triplicate, were run on a LightCycler 480 real-time PCR system and relative expression rations were calculated according to Pfaffl (2001).

### Dye-filling assay

Animals were washed off plates, washed three times with M9 buffer then incubated for one hour in 100 ng/μl Vybrant DiD cell-labeling solution (Invitrogen) as described previously (Schultz and Gumienny 2012). After destaining for one hour on NGMLite plates seeded with OP50 *E. coli*, animals were imaged by confocal microscopy at 40x magnification for DiD uptake (647nm excitation) in the amphid neurons and GFP expression (488nm excitation).

### Microscopy

Well-fed worms grown under standard conditions were used for expression pattern characterization. Images were captured on either a Zeiss LSM 700 confocal microscope, Zeiss Axiovision compound microscope, or Olympus BX61 compound microscope. Expression patterns in L1 larvae were imaged within 1 hr of hatching. For quantitative imaging of *cnd-1p*::*his-24*::*mCherry* expression in wild type and *cnd-1* mutant backgrounds, 2-4 cell embryos were isolated from gravid hermaphrodites and mounted on a bead pad (Murray *et al.* 2008). After 6-7 hr, when the embryos reached comma stage, a single image stack (to eliminate possible photobleaching) was captured for each embryo using a Zeiss LSM 700 confocal microscope at 40x magnification under identical settings, ensuring no detector saturation. Images were processed using Fiji (Schindelin *et al.* 2012) using a Z-project - Sum Slices workflow which rendered the summated stacks as 32-bit images. Pixel values and counts for the whole image (1024 × 1024 pixels) were obtained using the Analyze - Histogram function (using the pixel value range) then processed in Microsoft Excel (sum [pixel value x pixel count]).

Individual DD neuron identities was inferred by imaging *unc-25p*::*GFP* in L1 larvae then measuring the nose-to-neuron, neuron-to-neuron, or neuron-to-tail distances along the anterior-posterior body axis using the segmented line function in Fiji. DD neuron identity in *cnd-1**(**gk718**)* and *ceh-13**(**sw1**)* mutant combinations were mapped to the closest relative location in wild type L1 larvae. Total ventral nerve cord cells were determined by double-labeling with *juIs76** [unc-25p*::*GFP]* and *otIs356** [rab-3p(**prom1**)*::*2xNLS*::*TagRFP]* then counting from DD1 or the posterior end of the terminal pharyngeal bulb (if DD1 was absent) to the anus. In wild type, this included most of the 22 DA, DB and DD class motorneurons, plus some cells in the retrovesicular ganglion and tail region.

### Neuroanatomy

Cell identity was confirmed by crossing transgenic arrays into previously characterized strains then imaging as above. Table S1 shows the strains used for cell identification.

### Statistical analysis

Student *t*-tests were performed in Microsoft Excel, Mann-Whitney tests were performed in R, and two-tailed Fisher Exact tests were performed using GraphPad QuickCalcs. Graphs were generated in Excel or SAS. RNA-seq significance values reported in the text are not adjusted for false discovery. Benjamini-Hochberg corrections for multiple comparisons are available in the supplemental data. Bonferroni corrections were applied to other data where appropriate.

### Data availability

All reagents are available on request. Table S1 shows strains generated in this study. Tables S2 and S3 show lists of significantly down-regulated and up-regulated genes in the *cnd-1**(**ju29**)* comparative transcriptome. Figure S1 shows a volcano plot summarizing the *cnd-1**(**ju29**)* comparative transcriptome data. Figure S2 shows DEXseq hits identified in this work along with any expression validations. Figure S3 compares predicted CND-1 wild type and *ju29* mutant protein sequences. Figure S4 shows *cnd-1p*::*his-24*::*mCherry* and *unc-25p*::*GFP* co-localization. Raw and processed transcriptome files generated in this study are publicly available via the Gene Expression Omnibus (Edgar *et al.* 2002), accession number GSE125051 (https://www.ncbi.nlm.nih.gov/geo/query/acc.cgi?acc=GSE125051). Supplemental material available at figshare: https://doi.org/10.25387/g3.12567827.

## Results

### cnd-1 controls the expression of multiple genes During embryogenesis

Previous studies demonstrated that the proneural transcription factor *cnd-1* is active early in embryogenesis, with expression first being seen at the 14-cell stage and persisting until just prior to hatching (Hallam *et al.* 2000; Murray *et al.* 2012). *cnd-1* reporter gene expression decreases rapidly beyond the L1 stage but persists at low levels in adult head and ventral neurons (Kratsios *et al.* 2011). To gain a better understanding of how CND-1 controls gene expression during early nervous system development, we performed RNA-seq on RNA isolated from three samples each of N2 wild type and *cnd-1**(**ju29**)* mutant mixed stage embryos and analyzed the data using the DSeq2 package (Love *et al.* 2014). *cnd-1**(**ju29**)* is a G-to-A transition in the splice acceptor of intron 2 and behaves as a strong loss-of-function recessive allele (Hallam *et al.* 2000). *cnd-1**(**ju29**)* mutant embryos show 105 genes with significantly lower transcript levels (*P* < 0.05) when compared to wild type (Table S2) and 46 genes with significant higher transcript levels (Table S3, Figure S1). Table 1 shows the top 40 most significant hits sorted by up- *vs.* down-regulation and p-value. Surprisingly, only a single transcription factor gene, *ceh-5*, was identified in the down-regulated dataset, whereas three transcription factors genes (*nhr-68*, *nhr-77*, and *cnd-1* itself) were found in the up-regulated dataset. This suggests that *cnd-1* functions close to the end of a transcriptional regulatory cascade during *C. elegans* embryogenesis. This is in contrast to *ngn-1*/neurogenin, which controls expression of at least eight downstream transcription factors (Christensen *et al.* 2020).

**Table 1:** Top 40 differentially expressed genes in the *cnd-1(ju29)* RNA-seq dataset based on P-value. (A) Down-regulated genes; (B) up-regulated genes. Gene ID, gene identity; gene name, commonly used gene name/cosmid name; base mean, mean of normalized counts for that gene; log2 fold-change, log2 change in gene expression level when compared to wild type; P-value, significance; P-adj, significance with Benjamini-Hochberg adjustment for false discovery rate

A. *cnd-1(ju29)* transcriptome down-regulated genes (most significant p - value)					
Gene_ID	Gene Name	Base mean	log2 fold change	P-value	P-adj
WBGene00018031	*F35B3.4*	402	−1.7	6.5E-22	1.76E-17
WBGene00005832	*srw-85*	428	−1.2	3.7E-20	4.96E-16
WBGene00014955	*Y102A5C.6*	158	−1.0	2.6E-10	1.19E-06
WBGene00010212	*fbxa-192*	262	−0.9	2.6E-08	7.00E-05
WBGene00044213	*Y102A5C.36*	131	−0.9	1.2E-07	2.96E-04
WBGene00014954	*Y102A5C.5*	66	−0.9	7.1E-09	2.40E-05
WBGene00007454	*C08F11.7*	54	−0.9	3.6E-10	1.38E-06
WBGene00015990	*C18H2.3*	120	−0.9	1.3E-16	1.15E-12
WBGene00014454	*MTCE.7*	1917	−0.7	8.3E-06	0.016
WBGene00010958	*ndfl-4*	70	−0.7	8.4E-06	0.016
WBGene00010209	*fbxa-191*	31	−0.7	1.1E-08	3.38E-05
WBGene00007201	*exos-4.1*	947	−0.7	3.1E-05	0.056
WBGene00014472	*MTCE.33*	2190	−0.6	6.5E-04	0.93
WBGene00016953	*C55C3.3*	65	−0.5	1.1E-03	1.00
WBGene00015044	*cyp-34A9*	82	−0.5	1.0E-03	1.00
WBGene00016506	*abhd-5.1*	95	−0.5	1.9E-03	1.00
WBGene00006650	*tts-1*	1948	−0.5	2.8E-04	0.42
WBGene00000754	*col-181*	39	−0.5	3.1E-03	1.00
WBGene00000430	*ceh-5*	332	−0.5	6.2E-03	1.00
WBGene00014672	*C08F11.6*	27	−0.4	7.8E-04	1.00
WBGene00077585	*T01G5.8*	7	−0.3	5.7E-05	0.096
WBGene00202498	*Y60C6A.2*	35	−0.3	2.0E-03	1.00
WBGene00022013	*Y60C6A.1*	59	−0.3	2.1E-03	1.00
WBGene00002013	*hsp-12.6*	164	−0.2	4.4E-03	1.00
WBGene00012790	*Y43D4A.4*	6	−0.2	1.4E-04	0.22
WBGene00015549	*C06G3.3*	24	−0.2	2.9E-03	1.00
WBGene00008396	*D1086.9*	27	−0.2	7.2E-03	1.00
WBGene00017371	*sre-39*	4	−0.2	2.2E-03	1.00
WBGene00020178	*T02H6.8*	4	−0.1	3.5E-03	1.00
WBGene00045311	*Y57G11C.57*	3	−0.1	5.7E-03	1.00
WBGene00044293	*K08D12.7*	3	−0.1	6.2E-03	1.00
WBGene00044390	*ZK177.11*	3	−0.1	7.0E-03	1.00
WBGene00011429	*T04C12.7*	7	−0.1	7.8E-03	1.00

B. *cnd-1(ju29)* transcriptome up-regulated genes (most significant p - value)					
gene_ID	name	base mean	log2 fold change	P-value	P-adj
WBGene00018031	*lgc-34*	1214	1.3	4.30E-16	2.91E-12
WBGene00005832	*F35B3.3*	41	0.9	2.37E-11	1.28E-07
WBGene00014955	*ctc-3*	66934	0.7	6.72E-06	0.015
WBGene00010212	*ZC21.10*	882	0.5	0.003	1.00
WBGene00044213	*cnd-1*	958	0.5	0.004	1.00
WBGene00008677	*col-171*	8	0.1	0.007	1.00
WBGene00004567	*C32E8.4*	3	0.1	0.007	1.00

We also analyzed our data using DEXseq, a variant of the DSeq2 workflow (Anders *et al.* 2017). This analysis compares data sets by aligning individual sequence blocks (exons, alternative transcriptional start sites, and alternative splice sites) and is a sensitive way to identify splice or transcriptional variants between two datasets. Using this approach, *aak-2*, *srw-85*, and *ptrn-1* were found to have at least one significantly different transcript block in *cnd-1**(**ju29**)* mutants when compared to wild type (Figure S2). *aak-2* belongs to the AMP-activated protein kinase (AMPK) family and has roles in DAF-2-mediated insulin signaling, lifespan, and temperature-dependent dauer larva formation (Apfeld *et al.* 2004; Hardie 2014). In *cnd-1* mutants, the first exon of an internally transcribed *aak-2c* variant is expressed at a significantly higher level (*P* < 0.05), suggesting that CND-1 may repress this internally transcribed variant in wild type animals (Figure S2A).

DEXseq identification of *ptrn-1* transcript differences were resolved by visual inspection of the *ptrn-1* genomic locus using Integrated Genome Viewer (Robinson *et al.* 2011; Thorvaldsdottir *et al.* 2013). This revealed loss of all gene transcription in the 7-8kb region immediately downstream of *ptrn-1*. Genomic PCR coupled with Sanger sequencing confirmed this to be a novel 6906bp deletion/ 80bp insertion allele that removes most of the *ptrn-1* 3′ UTR including the poly-adenylation signal, along with three downstream genes (F35B3.1, F35B3.4 and F35B3.10), of which F35B3.1 and F35B3.4 were also differentially expressed in our transcriptome (Figure S2I-L and Table S2). F35B3.10 codes for a predicted snRNA whose transcript was not represented in either wild type or *cnd-1**(**ju29**)* datasets. *ptrn-1* codes for a known neuronal microtubule stabilizing protein (Chuang *et al.* 2014; Marcette *et al.* 2014; Richardson *et al.* 2014), so it is possible that this *ptrn-1**(**ken2**)* deletion may enhance the *cnd-1**(**ju29**)* uncoordinated phenotype and be selected for during out-cross. To control for this possibility, we performed validation assays using the *cnd-1**(**gk718**)* mutation, which is a large deletion allele, predicted to be a null mutant, and was shown by genomic PCR not to contain the *ken2* deletion (Figure S2K).

### cnd-1 controls ceh-5 expression in a subset of neurons

**Figure 1:**
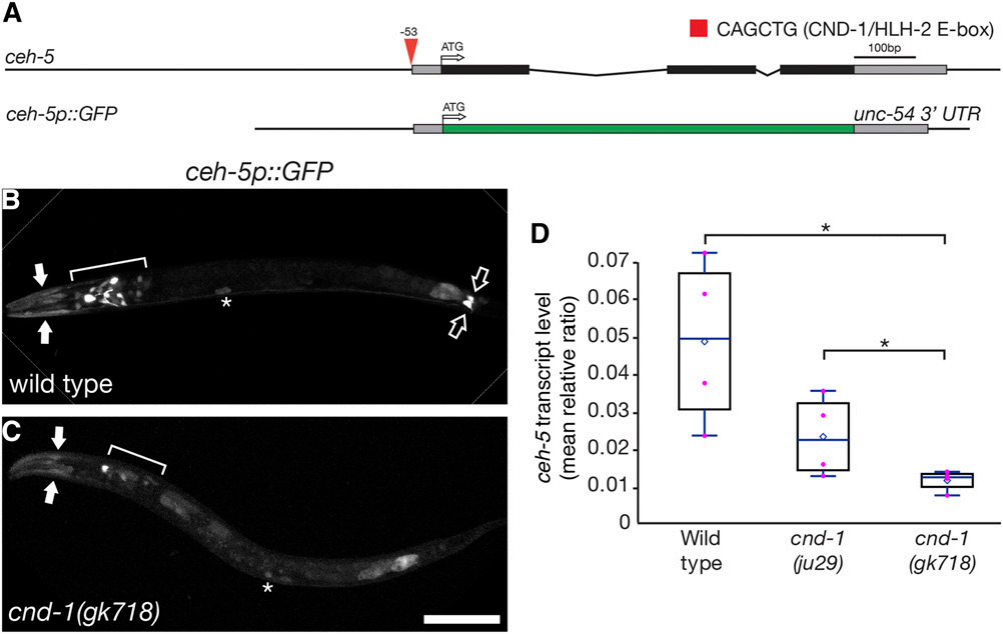
*cnd-1* controls *ceh-5* expression in a subset of neurons. (A) Schematic of the *ceh-5* genomic region showing predicted CND-1/HLH-2 binding site (Grove *et al.* 2009), and structure of the *ceh-5* reporter gene used in this study. (B, C) *ceh-5p*::*GFP* reporter gene expression in wild type (B) and *cnd-1**(**gk718**)* mutants (C). Filled arrows, head muscles; open arrows, PVQL/R neurons; asterisks, coelomocytes. Bracketed regions show RME plus other head neurons. Scale bar = 25μm. (D) Box and whisker plot showing average quantitative RT-PCR levels of *ceh-5* mRNA transcript in wild type, *cnd-1**(**ju29**)* and *cnd-1**(**gk718**)* mutants. Open diamond, average; box shows median, first, and third quartiles. Whiskers show data extremes in 1.5 × interquartile range. Data are relative to *cdc-42* mRNA. * *P* < 0.025, Student’s *t*-test with Bonferroni correction for multiple comparisons.

Our comparative transcriptome showed that *ceh-5* was significantly down-regulated in *cnd-1**(**ju29**)* mutants when compared to wild type (Table 1). *ceh-5* is the *C. elegans* ortholog of the mammalian transcription factor ventral anterior homeobox 2 gene (Vax2), which is required for correct dorsoventral patterning of the eye (Take-uchi *et al.* 2003; Liu *et al.* 2008; Alfano *et al.* 2011). A sequence search across the *ceh-5* locus found a single candidate *cnd-1* CATATG E-box binding site about 50bp 5′ to the *ceh-5* translational start site (Figure 1A). To better understand the role of *cnd-1* in controlling *ceh-5* expression, we used a *ceh-5p*::*GFP* reporter gene to compare expression patterns in wild type and *cnd-1* mutants (Figure 1, Table 2) (Reece-Hoyes *et al.* 2007). In wild type L1 larvae, *ceh-5p*::*GFP* showed robust expression in head muscles, a subset of head neurons (including the RME neurons), and five or six cells in the tail including the PVQL/R neurons (Figure 1B). Weak *ceh-5p*::*GFP* expression was also seen in the coelomocytes (asterisks, Figure 1B) and the pharyngeal terminal bulb. In *cnd-1**(**gk718**)* mutants, *ceh-5p*::*GFP* expression was lost from many head neurons including the RMEs, and also the tail neurons (Figure 1C), but was retained in some head muscles and also the coelomocytes. We used quantitative PCR to further validate *ceh-5* transcript levels; these were significantly lower in both *cnd-1**(**ju29**)* and *cnd-1**(**gk718**)* RNA samples, with *gk718* showing lower *ceh-5* transcript levels when compared to *ju29*, suggesting that CND-1(ju29) may retain some function (Figure 1D). Together, these data indicate that CND-1 is necessary for *ceh-5p*::*GFP* expression in a subset of cells including the RME head and PVQ tail neurons, although it is not known if these cells are lost, changing fate, or are merely losing *ceh-5* reporter gene expression.

**Table 2:** Summary of *ceh-5p*::*GFP* expression in L1 larvae. (A) Average number of head neurons, head muscle bundles, and tail neurons observed in wild type and *cnd-1(gk718)* mutants respectively (+/− standard error of the mean). ** *P* < 0.01, Mann-Whitney *U*-test with continuity correction. (B) Percentage of animals showing expression in other tissues. Note that the GFP reporter stain used in this assay was an extra-chromosomal array and showed some expression variability between animals. ** *P* < 0.01, Fisher Exact test

A. *ceh-5p*::*GFP* expression in neurons and head muscles			
Strain	# head neurons	# head muscles	# tail neurons
wild type (n = 11)	12.5 (1.6)	4.0 (0)	2.2 (0.3)
*cnd-1(gk718)* (n = 5)	4.8 (0.8)**	2.2 (0.7)**	0.0 (0)**

B. *ceh-5p*::*GFP* expression in other tissues			
Strain	pharynx	coelomocytes	gut
wild type (n = 11)	100%	73%	100%
*cnd-1(gk718)* (n = 5)	20%**	60%	100%

### CND-1 controls the fate of some cnd-1-expressing cells during embryonic nervous system development

**Figure 2:**
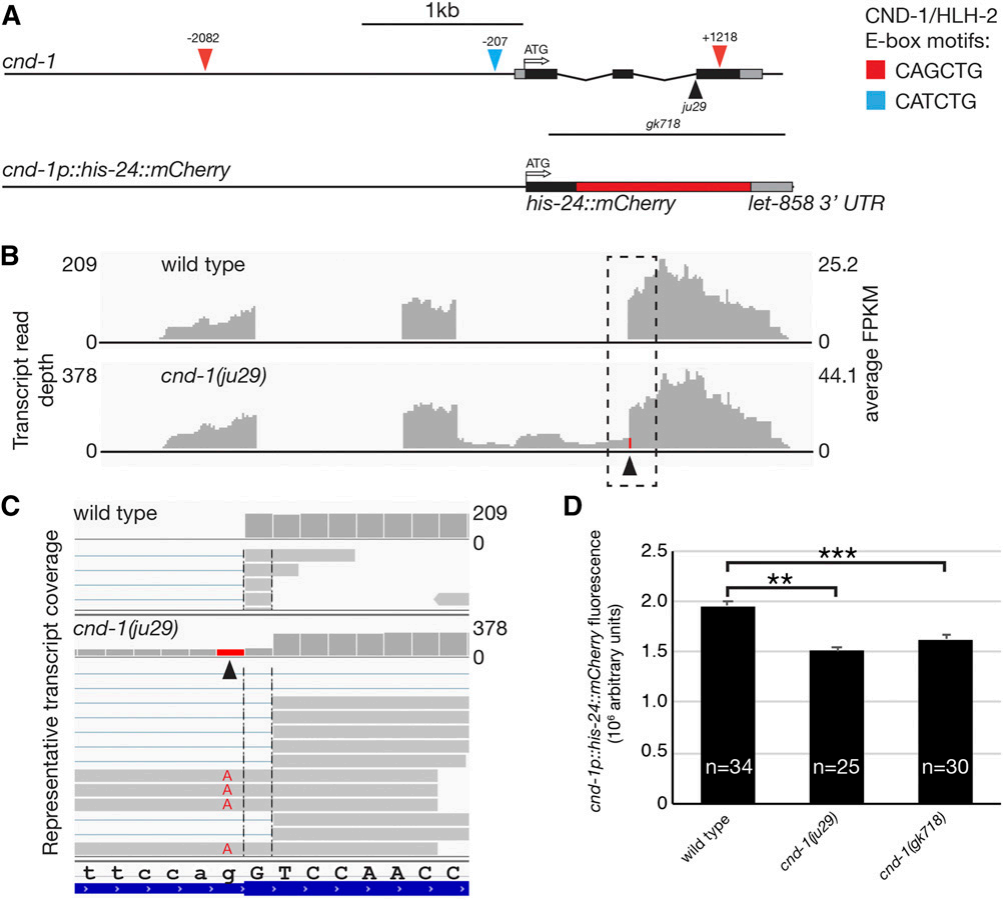
*cnd-1**(**ju29**)* transcript levels are significantly up-regulated when compared to wild type, although embryonic *cnd-1p*::*mCherry* fluorescence is reduced in *cnd-1* mutants. (A) Schematic of the *cnd-1* genomic region showing location of predicted CND-1/HLH-2 binding sites, and the *stIs10055** [cnd-1p*::*his-24*::*mCherry]* reporter gene used extensively in this study. (B) Integrated Genome Viewer output of wild type and *cnd-1**(**ju29**)* RNA-seq reads showing raw transcript depth and average Fold Per Kilobase Million coverage. Arrowhead shows location of the *ju29* mutation. (C) Inset of boxed region in (B), showing representative reads, the *ju29* G-to-A mutation, and the non-canonical 3′ splice acceptor used in the *ju29* mutant. (D) Quantitative fluorescence of *cnd-1p*::*his-24*::*mCherry* expression in comma-stage embryos in wild type, *cnd-1**(**ju29**)* and *cnd-1**(**gk718**)* mutant embryos. Error bars show standard error of the mean. ** *P* < 0.005, *** *P* < 0.0005, Student’s *t*-test with Bonferroni correction for multiple comparisons.

The *cnd-1* locus is comprised of three exons spanning a 1.5kb region of chromosome III (Figure 2A). As mentioned previously, the *cnd-1**(**ju29**)* allele used in our transcriptome analysis is a G-to-A transition in the splice acceptor of intron 2 and was predicted to force a splice onto a non-canonical splice acceptor leading to a frame shift (Hallam *et al.* 2000). When *cnd-1* RNA-seq data were viewed using Integrated Genome Viewer, we confirmed that *cnd-1**(**ju29**)* transcripts showed the G-to-A transition at the *ju29* base change and also a 1bp shift in the splice acceptor (Figure 2B, red column at the start of exon 3; Figure 2C; Figure S3). In addition, around 20% of transcripts show inclusion of intron 2, presumably because the *ju29* mutation creates a weak splice acceptor. Figure 2B shows representative read depth across the *cnd-1* locus. In terms of raw reads and when normalized to Fragments per Kilobase Million (FPKM), *cnd-1* transcript levels are almost twice as high in *cnd-1**(**ju29**)* compared to wild type, suggesting that CND-1 may be partially responsible for regulating its own transcript levels via transcriptional repression.

To further explore these data, we performed quantitative confocal microscopy on wild type and *cnd-1* mutant comma stage embryos carrying an integrated *cnd-1p*::*his-24*::*mCherry* transcriptional reporter gene (Murray *et al.* 2012). The comma stage is easily identified during embryonic development and provides a defined time point to quantitatively compare mCherry expression levels. Contrary to what RNA-seq revealed, we found that *cnd-1p*::*mCherry* reporter gene expression was significantly lower in *cnd-1* mutants when compared to wild type (Figure 2D, *P* < 0.005, n = 34, 25, and 30 embryos analyzed for wild type, *cnd-1**(**ju29**)* and *cnd-1**(**gk718**)* mutants respectively). The reason for this discrepancy was unclear but may be assay dependent. For instance, inclusion of intron 2 within the *cnd-1**(**ju29**)* mRNA transcript may increase its representation within the RNA-seq dataset, causing it to appear up-regulated. Alternatively, it may increase the stability of *cnd-1**(**ju29**)* transcripts, causing an apparent up-regulation of RNA levels.

**Figure 3:**
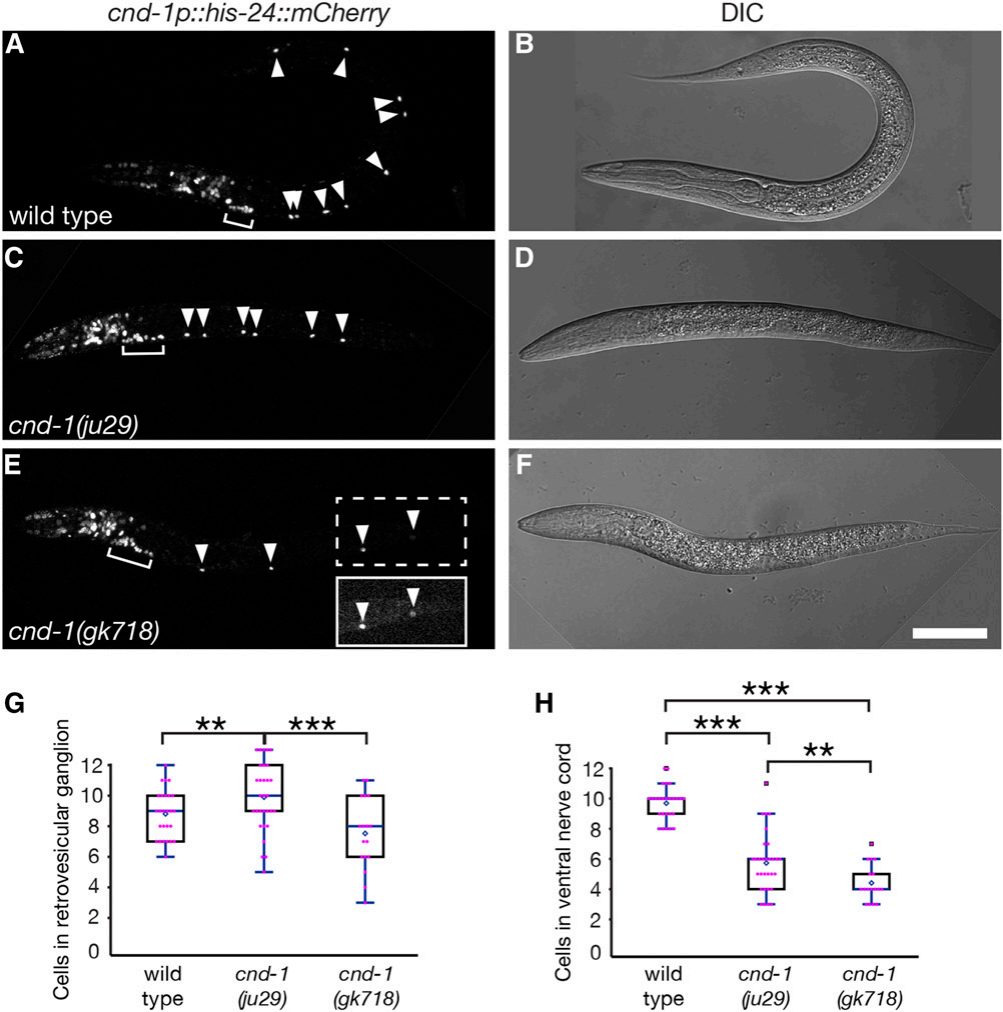
*cnd-1* controls the fate of some *cnd-1*-expressing cells in the retrovesicular ganglion and ventral nerve cord. (A-F) Confocal and DIC micrographs of *cnd-1p*::*his-24*::*mCherry* expression in (A) wild type, (C) *cnd-1**(**ju29**)*, and (E) *cnd-1**(**gk718**)* mutants. (B, D, F) DIC images of the above. Arrowheads show *cnd-1*-positive nuclei in the ventral nerve cord. Bracket shows retrovesicular ganglion. Inset in (E) is contrast-stretched to highlight the two most posterior *cnd-1*-positive neurons. Scale bar in (F) = 25μm. (G, H) Box and whisker plots showing average number of *cnd-1*-positive cells in the retrovesicular ganglion and ventral nerve cord respectively. n = 26, 19, and 17 animals for wild type, *cnd-1**(**ju29**)*, and *cnd-1**(**gk718**)* respectively. Open diamond shows the average; box shows median, first, and third quartiles; whiskers show data extremes in 1.5 × interquartile range with outliers shown beyond. ** *P* < 0.005; *** *P* < 0.0005, Mann-Whitney *U*-test with continuity correction and Bonferroni correction for multiple comparisons.

To clarify the role of *cnd-1* during nervous system development, we also analyzed *cnd-1* reporter gene expression in L1 larvae. Only three types of ventral cord motorneuron (cholinergic DA and DB, and GABAergic DD class) are born during embryogenesis and can be easily assayed in young L1 larvae using genetically encoded reporter genes. Previous data showed that *cnd-1**(**ju29**)* mutants exhibit variable loss of all three embryonic motor neuron types (Hallam *et al.* 2000). We examined *cnd-1p*::*mCherry* expression in early L1 larvae, within an hour of hatching, counting nuclei in the retrovesicular ganglion and also the ventral nerve cord (Figure 3). The retrovesicular ganglion is a linear cluster of cells located on the ventral midline of the worm immediately posterior to the pharynx, and contains the anterior most DA, DB and DD motorneurons (DA1, DB1, DB2 and DD1), along with eight additional cells. Wild type animals showed an average of nine *cnd-1p*::*mCherry* nuclei in the retrovesicular ganglion and 10 in the ventral nerve cord (n = 26). As there are 22 motorneurons in early L1 larvae, this suggests that only a subset express the *cnd-1* reporter gene. Based on their location along the ventral nerve cord, co-labeling with *unc-25p*::*GFP* (a known DD neuron marker), and by corroborating against single cell RNA-seq expression data (Packer *et al.* 2019), we tentatively conclude that *cnd-1p*::*mCherry* is expressed in DA1-5, DB1, DB3, and DD1-6 (Table 3 and Figure S4). In *cnd-1**(**ju29**)* mutants, the average number of *cnd-1*-positive cells in the retrovesicular ganglion increased to 10 (*P* < 0.005), although the number of cells in the ventral nerve cord dropped dramatically to around six (n = 19, *P* < 0.001). In *cnd-1**(**gk718**)* mutants, the number of *cnd-1*-positive cells dropped to an average of seven in the retrovesicular ganglion and four in the ventral nerve cord (*P* < 0.0005). When comparing *cnd-1p*::*mCherry* expression between the *cnd-1**(**ju29**)* and *cnd-1**(**gk718**)* backgrounds, both retrovesicular ganglion and ventral nerve cord cell counts are significantly different (*P* < 0.0005 for retrovesicular ganglion cells, and *P* < 0.005 for ventral nerve cord cells). Based on these reporter gene studies, we conclude that CND-1 is required for the fate specification of a subset of embryonic ventral nerve cord neurons, confirming data reported by Hallam *et al.* (2000). In addition, the above data suggests that the CND-1(ju29) protein retains some activity, or in some contexts behaves in a neomorphic manner to affect the developmental outcome of CND-1-dependent cell fates (Figure S4). For this reason, all remaining analyses were performed using the *cnd-1**(**gk718**)* allele.

**Table 3:** *cnd-1* and *unc-25* markers co-express in sub-sets of ventral cord motorneurons. N = 21 wild type animals scored. Cells are listed from left-to-right in the anterior-to-posterior order they appear in L1 larvae. *In two animals, we saw an additional cell body (tentatively identified as RIGL) between DB3 and DA2. ^#^Three animals had *cnd-1*-positive cells at the DB5 location. However, we suspect these are animals where DA4 and DB5 switched position during development

Ventral cord neuron	DB2	DD1	DB1	DA1	DB3	RIGL*	DA2	DD2	DA3	DB4	DA4	DD3
*unc-25p*::*GFP*	0	21	0	0	0	0	0	21	0	0	0	21
*cnd-1p*::*mCherry*	0	21	20	21	18	2	21	21	21	0	18	21

Ventral cord neuron	DB5	DA5	DD4	DB6	DA6	DD5	DB7	DA7	DD6	DA8	DA9	
*unc-25p*::*GFP*	0	0	21	0	0	21	0	0	21	0	0	
*cnd-1p*::*mCherry*	3^#^	21	21	0	0	21	0	0	21	0	0	

### cnd-1 and ceh-13 are co-expressed in a subset of ventral cord motorneurons

**Figure 4:**
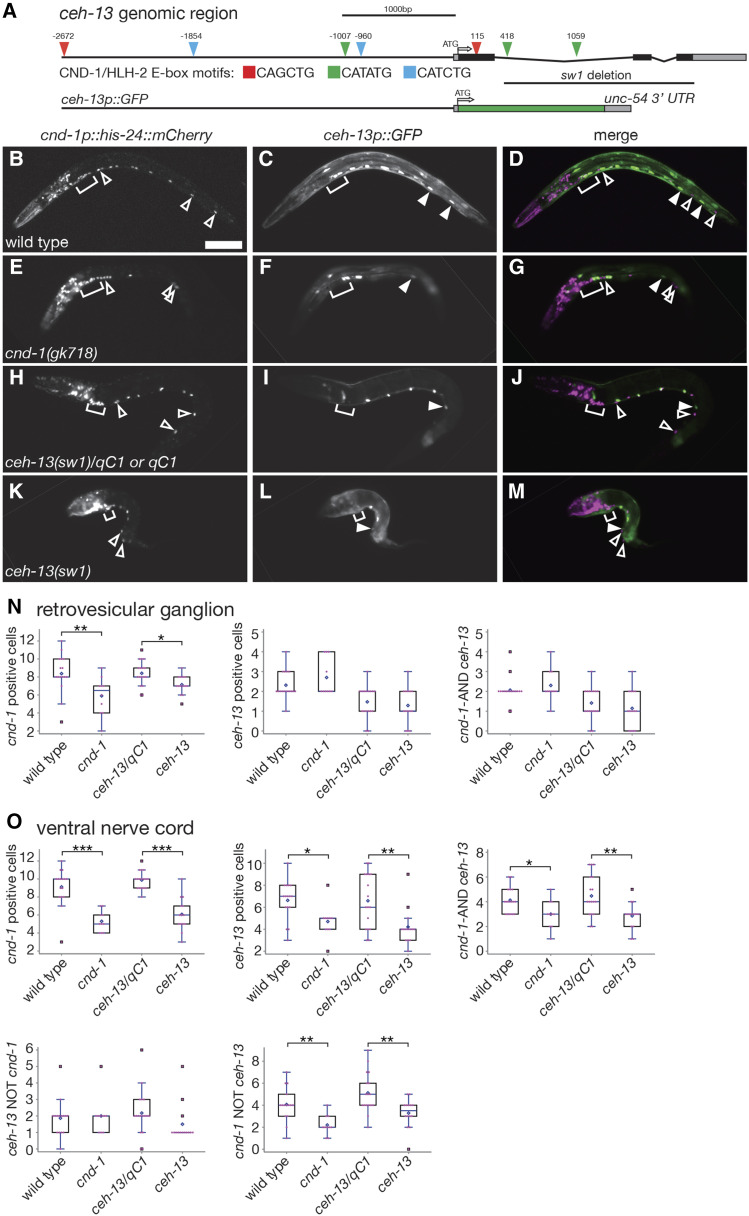
*ceh-13* and *cnd-1* show partially overlapping expression in the ventral nerve cord. (A) Schematic of the *ceh-13* genomic region showing predicted CND-1/HLH-2 binding sites, location of the *ceh-13**(**sw1**)* mutation, and structure of the *ceh-13* reporter gene used in this study. (B-M) Expression patterns of *cnd-1p*::*his-24*::*mCherry* and *ceh-13p*::*GFP* in wild type, *cnd-1**(**gk718**)*, *ceh-13**(**sw1**)/**qC1* (or *qC1*), and *ceh-13**(**sw1**)* mutants. while most VNC cells express both *cnd-1* and *ceh-13*, a distinct subset does not, and multiple cells are absent in both *cnd-1* and *ceh-13* mutants. Open arrowheads, *cnd-1* expressing cells only; filled arrowheads, *ceh-13* expressing cells only; bracket, retrovesicular ganglion. Scale bar in (B) = 25μm. (N, O) Box and whisker plots of *cnd-1* and *ceh-13* reporter genes showing average cell counts in the retrovesicular ganglia and ventral nerve cord respectively. Open diamond shows the average; box shows median, first, and third quartiles; whiskers show data extremes in 1.5 × interquartile range with outliers shown beyond. * *P* < 0.05; ** *P* < 0.01; *** *P* < 0.001, Mann-Whitney *U*-test with continuity correction.

Previous work using RNA-seq analysis of FACS-isolated *cnd-1p*::*mCherry*-positive cells showed that Hox gene *ceh-13*/labial transcripts were enriched in *cnd-1*-expressing cells when compared to negative controls (Burdick *et al.* 2016). The *ceh-13* locus has multiple consensus CND-1 E-box binding sites (Figure 4A), raising the possibility that *cnd-1* may control aspects of *ceh-13* transcription. In addition, *ceh-13* mutants exhibit loss of ventral cord motorneurons similar to that seen in *cnd-1* mutants (Stefanakis *et al.* 2015). This led us to investigate the relationship between *cnd-1* and *ceh-13* in controlling embryonic motorneuron cell fate specification. We examined *ceh-13p*::*GFP* and *cnd-1p*::*mCherry* expression in the retrovesicular ganglion and ventral nerve cords of wild type, *cnd-1**(**gk718**)*, *ceh-13**(**sw1**)/**qC1* heterozygotes, and *ceh-13**(**sw1**)* homozygous mutant L1 larvae (Figure 4B-G). *ceh-13**(**sw1**)* is a 1.5kb deletion of that removes most of intron 1, and all of exons 2 and 3 (Figure 4A) and behaves as a recessive null allele (Brunschwig *et al.* 1999). *ceh-13**(**sw1**)* homozygous animals are 97% embryonic lethal, with the remaining 3% of surviving larvae showing strong body morphology defects. We used this phenotype to identify L1 stage *sw1* homozygotes to analyze for *ceh-13p*::*GFP* and *cnd-1p*::*mCherry* expression. In wild type and *ceh-13**(**sw1**)/**qC1* heterozygotes, *cnd-1p*::*mCherry* and *ceh-13p*::*GFP* showed a complex and partially overlapping expression pattern in the retrovesicular ganglion and ventral nerve cord, with around two cells co-expressing *cnd-1* and *ceh-13* in the ganglion and an average of 4.1 (wild type) and 4.7 cells (*ceh-13**/**qC1**)* co-expressing in the ventral nerve cord (Figure 4B-O, Tables 4 and 5). In *cnd-1**(**gk718**)* and *ceh-13**(**sw1**)* homozygotes, the average number of cells co-expressing each marker in the ventral nerve cord dropped significantly to 3.0 and 2.8 for *cnd-1* and *ceh-13* respectively. Both *cnd-1* and *ceh-13* homozygous mutants also showed a significant reduction in cells that expressed *cnd-1p*::*mCherry* only, but not *ceh-13p*::*GFP*. Based on the similarity in phenotypes shown, we conclude that both *cnd-1* and *ceh-13* have roles in controlling a subset of ventral nerve cord cell fates during embryogenesis. We note that the ganglion and ventral cord cell counts in this double reporter gene assay were slightly different from the data reported in Figure 3. However, the data in Figure 3 was captured on a confocal microscope whereas the data in Figure 4 was captured using epifluorescence, which gives slightly lower resolution and may have led to an undercount of cells that were directly adjacent to or behind each other.

**Table 4:** *cnd-1* and *ceh-13* markers co-express in mid-body DA and DD ventral cord motorneurons. Percentage of *cnd-1p*::*mCherry* and *ceh-13p*::*GFP*-positive neurons scored each for ventral nerve cord cell assayed. N = 10 wild type animals scored. Cells are listed from left-to-right in the anterior-to-posterior order they appear in L1 larvae. Strains that were highly mosaic for the *ceh-13p*::*GFP* reporter gene were omitted from this analysis

	DA2	DD2	DA3	DA4	DD3	DA5	DD4	DA6	DD5	DA7	DD6
*cnd-1p*::*mCherry*	100	100	100	100	100	100	100	0	100	0	100
*ceh-13p*::*GFP*	50	50	80	90	60	100	40	90	0	80	0

**Table 5:** Summary of *cnd-1p*::*mCherry* and *ceh-13p*::*GFP* expression in L1 larvae. (A, B) Average number of cells showing reporter gene expression in the retrovesicular ganglion and ventral nerve cord respectively (+/− standard error of the mean). * *P* < 0.05; ** *P* < 0.01, *** *P* < 0.001, Mann-Whitney *U*-test with continuity correction (*cnd-1* compared against wild type and *ceh-13* compared against *ceh-13/qC1*). The *ceh-13p*::*GFP* reporter stain used in this assay was an extra-chromosomal array and showed some expression variability between animals

A. Cells in retrovesicular gangion						
	N	*cnd-1p*:: *mCherry*	*ceh-13p*:: *GFP*	*cnd-1* AND *ceh-13*	*ceh-13* NOT *cnd-1*	*cnd-1* NOT *ceh-13*
wild type	16	8.4(0.5)	2.3(0.2)	2.1(0.2)	—	—
*cnd-1(gk718)*	10	5.9(0.7) **	2.7(0.3)	2.3(0.3)	—	—
*ceh-13(sw1)/qC1 or qC1*	17	8.5(0.4)	1.3(0.2)	1.3(0.2)	—	—
*ceh-13(sw1)*	14	6.9(0.3) *	1.2(0.3)	1.0(0.3)	—	—
B. Cells in ventral nerve cord						
wild type	16	9.1(0.5)	6.6(0.4)	4.1(0.3)	1.9(0.3)	4.1(0.4)
*cnd-1(gk718)*	10	5.3(0.4) ***	4.7(0.5) *	3.0(0.4) *	2.0(0.4)	2.2(0.3) **
*ceh-13(sw1)/qC1 or qC1*	17	10.0(0.2)	6.5(0.6)	4.7(0.4)	1.4(0.4)	4.8(0.5)
*ceh-13(sw1)*	14	5.8(0.4) ***	4.2(0.5) **	2.8(0.3) **	1.6(0.3)	3.3(0.4) **

### cnd-1 and ceh-13 function redundantly to induce DD1 and DD2 motorneuron fate

**Figure 5:**
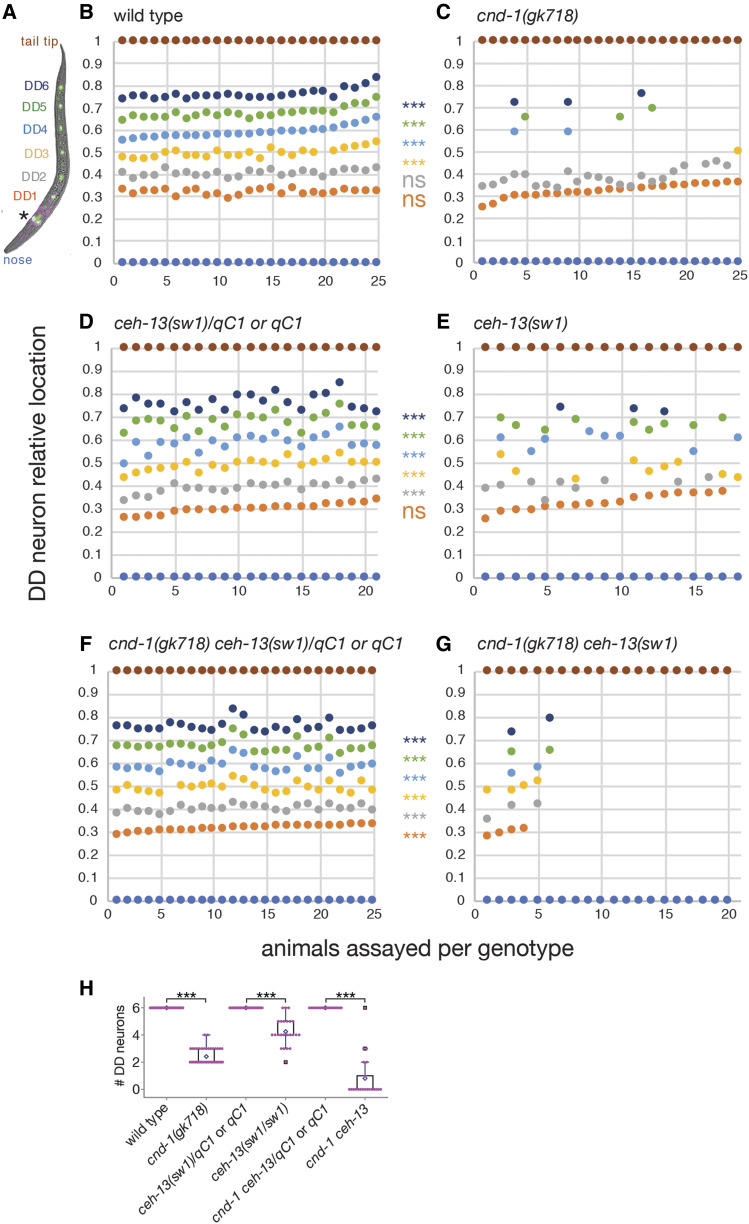
*cnd-1* and *ceh-13* function redundantly to control a subset of DD motorneuron cell fates. (A) Representative image of *unc-25p*::*GFP* and *cnd-1p*::*his-24*::*mCherry* expression in an L1 larva showing DD neuron color key. Asterisk shows RME head neurons. (B-G) Plots of DD neuron relative location in (B) wild type, (C) *cnd-1**(**gk718**)*, (D) *ceh-13**(**sw1**)/**qC1*, (E) *ceh-13**(**sw1**)*, (F) *cnd-1**(**gk718**) **ceh-13**(**sw1**)/**qC1*, and (G) *cnd-1**(**gk718**) **ceh-13**(**sw1**)* L1 larvae. Body morphology defects were used to identify *sw1* or *gk718* *sw1* homozygous animals. *** *P* < 0.001, Fisher exact test, presence *vs.* absence of each DD neuron type between control and experimental group (ns = not significant). (H) Box and whisker plots showing average number of DD neurons in the above strains. Open diamond shows the average; box shows median, first, and third quartiles; whiskers show data extremes in 1.5 × interquartile range with outliers shown beyond. *** *P* < 0.001, Mann-Whitney *U*-test with continuity correction.

The similarity in *cnd-1* and *ceh-13* loss-of-function phenotypes and their effect on each other’s reporter gene expression suggest that they may function together, either to cross-regulate each other or to specify ventral nerve cord motor neuron fate. To clarify this, we used an *unc-25p*::*GFP* reporter gene (Jin *et al.* 1999) to examine DD motorneuron fate in *cnd-1**(**gk718**)* and *ceh-13**(**sw1**)* single mutants, and *cnd-1**(**gk718**) **ceh-13**(**sw1**)* double mutant L1 larvae (Figure 5). *unc-25p*::*GFP* is expressed in the six DD neurons (annotated in color, Figure 5A), in addition to the four RME head neurons (asterisk, Figure 5A). We also plotted the cell body location of each DD neuron relative to the nose and tail tip, to establish which cells were more sensitive to loss of *cnd-1* or *ceh-13*. Wild type animals showed GFP expression in all six DD neurons (Figure 5B, H), in agreement with previous studies (Jin *et al.* 1999). However, *cnd-1**(**gk718**)* mutants showed an average of 2.5 DD neurons (Figure 5C, H, n = 25, *P* < 0.001), with DD1 and DD2 being retained and DD3-6 being lost. L1 larvae of *ceh-13**(**sw1**)/**qC1* or *qC1* genotype (*i.e.*, those with wild type morphology) showed GFP expression in all 6 DD neurons (n = 21 larvae scored). In contrast, *ceh-13**(**sw1**)* homozygous animals (identified by body morphology defects) showed on average four DD neurons, with DD1 generally being present but with variable loss of DD2-6 (Figure 5E, H, n = 18, *P* < 0.001). *cnd-1* *ceh-13**/**qC1* balanced double mutants again showed a wild type DD neuron induction pattern consistent with both *cnd-1* and *ceh-13* displaying recessive phenotypes (Figure 5F, H). However, 12/20 *cnd-1**(**gk718**) **ceh-13**(**sw1**)* homozygous double mutants showed no DD neuron fate induction, with the remaining animals showing variable induction of one or more DD neurons (Figure 5G, H, n = 20, *P* < 0.001). One animal with body morphology defects had six DD neurons. We speculate that this may have been a *cnd-1* *ceh-13**/**qC1* heterozygous or *qC1* homozygous animal that happened to have a body morphology defect. Overall, this suggests that *cnd-1* is primarily required for fate induction of DD3 through DD6, with *ceh-13* playing only a minor role in this process. In contrast, *cnd-1* and *ceh-13* have redundant roles in DD1 and probably DD2 fate induction, with loss of either transcription factor still allowing robust fate specification. Note that distinguishing between DD2 and DD3 neurons was difficult in some animals, so DD2 fate is likely to be under-counted in *ceh-13* mutants.

### cnd-1 and ceh-13 are redundantly required for setting ventral nerve cord cell number

**Figure 6:**
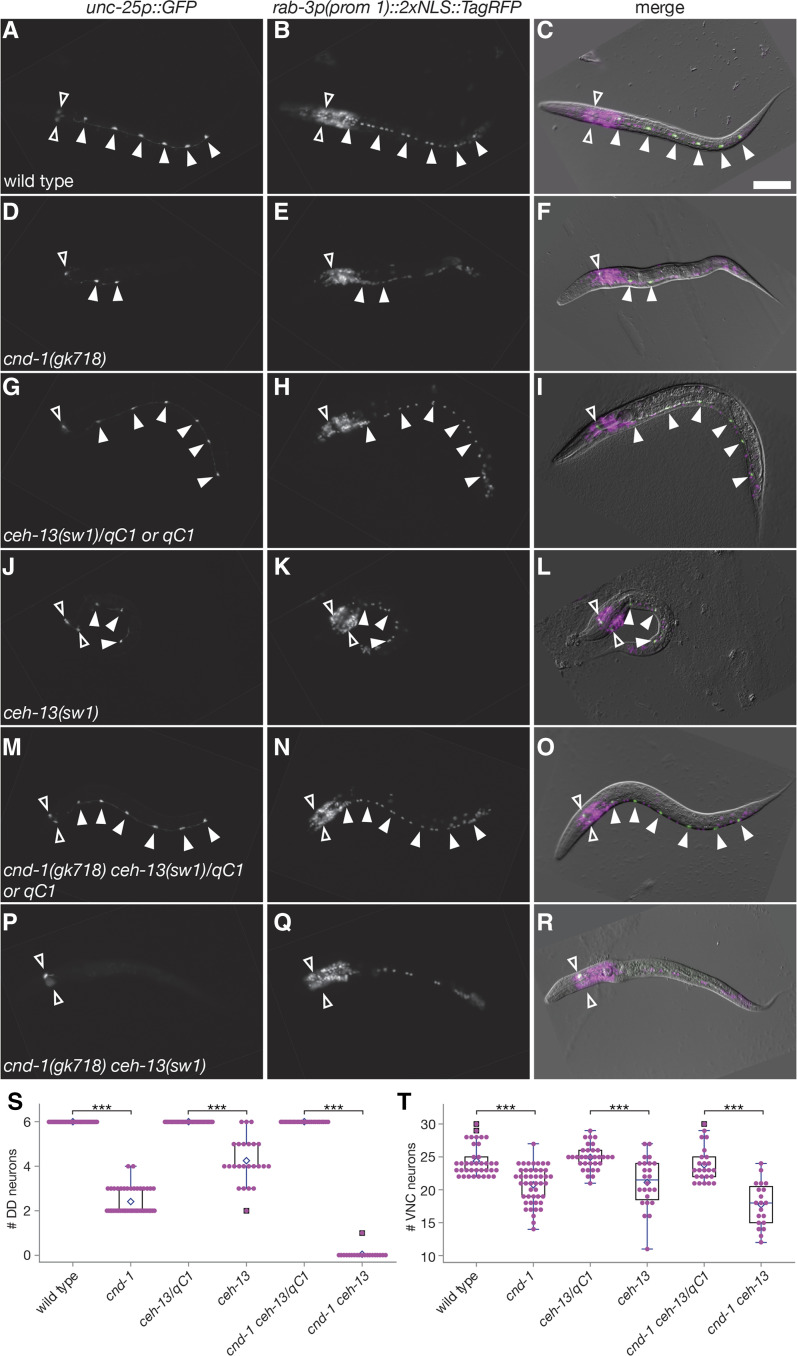
*cnd-1* and *ceh-13* control the birth of DD motorneurons but have no obvious role in DA or DB motorneuron birth. (A-R) Representative images of DD neurons (identified by *unc-25p*::*GFP* expression) and all ventral cord motorneurons (identified by *rab-3p(prom 1)*::*2xNLS*::*TagRFP* expression) in (A-C) wild type, (D-F) *cnd-1**(**gk718**)*, (G-I) *ceh-13**(**sw1**)/**qC1*, (J-L) *ceh-13**(**sw1**)*, (M-O) *cnd-1**(**gk718**) **ceh-13**(**sw1**)/**qC1*, and (P-R) *cnd-1**(**gk718**) **ceh-13**(**sw1**)* L1 larvae. Body morphology defects were used to identify *sw1* or *gk718* *sw1* homozygous animals. Filled arrowheads show DD motor neurons; open arrowheads show RME head neurons, which also express the *unc-25p*::*GFP* reporter. Scale bar in (C) = 25μm. (S, T) Box and whisker plots showing average number of (S) DD neurons and (T) all ventral cord motorneurons in the above strains. Open diamond shows the average; box shows median, first, and third quartiles; whiskers show data extremes in 1.5 × interquartile range with outliers shown beyond. *** *P* < 0.001, Mann-Whitney *U*-test with continuity correction.

Our data above indicate that both *cnd-1* and *ceh-13* have roles in regulating expression of *unc-25p*::*GFP*, a reporter gene for GABAergic terminal fate specification of DD motorneurons. However, loss of *unc-25p*::*GFP* expression does not necessarily mean loss of neural fate induction. For instance, DA2-DA5 cholinergic motorneurons share the same great-grandmother cell that gives rise to the DD neurons, raising the possibility that DD cells might switch fates in *cnd-1* or *ceh-13* mutants (Sulston *et al.* 1983). To address this possibility, we examined DD fate induction using *unc-25p*::*GFP* in the presence of pan-neuronal terminal fate marker *rab-3p(prom1)*::*2xNLS*::*TagRFP* (Stefanakis *et al.* 2015). Wild type animals (n = 37) showed an average of 24 ventral nerve cord cells, of which six were *unc-25p*::*GFP*-positive consistent with our previous data (Figure 6A - C). However, in *cnd-1**(**gk718**)* mutants (n = 46), only 20 ventral cord cells were observed (Figure 6D - F, *P* < 0.001 compared to wild type). In addition, they showed a parallel loss of around four DD cells similar to Figure 5C and 5H. *ceh-13**(**sw1**)* homozygous mutants (n = 24) showed similar phenotypes, with 21 ventral cord cells counted compared to 25 in *sw1**/**qC1* balanced heterozygotes (n = 33) (Figure 6G - L, *P* < 0.001). Finally, *cnd-1**(**gk718**) **ceh-13**(**sw1**)* double homozygous mutants (n = 20) showed around 18 cells in the ventral cord when compared to an average of 24 cells in *gk718* *sw1**/**qC1* balanced heterozygotes (n = 24) (Figure 6M - R). Figure 6S and 6T summarize average DD neuron and ventral cord motorneuron counts for the above assays. Overall, the difference in ventral cord neuron count parallels the loss of DD neurons observed in *cnd-1* and *ceh-13* mutant backgrounds and argues against a change of DD neuron fate to another neuronal cell type.

## Discussion

### The role of cnd-1 as a proneural transcription factor

**Figure 7:**
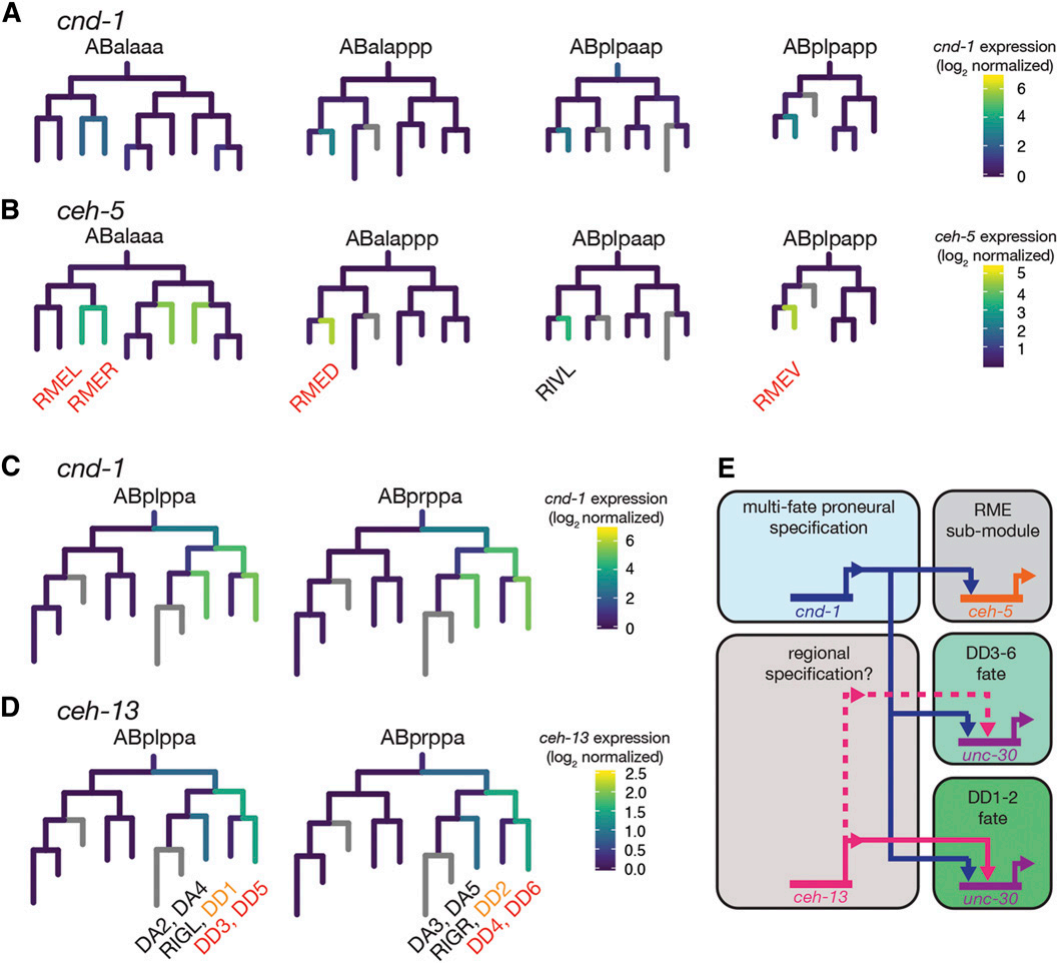
Summary of *cnd-1**’s* role in controlling a *ceh-5*-dependent RME sub-module and DD motorneuron fate. (A - D) Single-cell RNA-seq expression lineages of (A, C) *cnd-1*, (B) *ceh-5*, and (D) *ceh-13* expression. Data derived from Packer *et al.* (2019) and visualized using the Viscello data display tool (https://cello.shinyapps.io/celegans/). Co-expression of *cnd-1* and *ceh-5* transcripts occurs in all RME-class neurons. We find that *ceh-5p*::*GFP* expression in RME neurons is lost in *cnd-1**(**gk718**)* mutants suggesting that *cnd-1* is responsible for driving *ceh-5* expression in these cells. Based on the above expression overlap, we predict that *cnd-1* also controls *ceh-5p*::*GFP* expression in RIVL/R. Note that *ceh-5* does not control RME neuron fate, because those cells can still be visualized using an *unc-25p*::*GFP* reporter gene. Similarly, *cnd-1* and *ceh-13* are co-expressed in DD3-6 motorneurons but not in DD1 and DD2. *unc-25p*::*GFP* expression in DD3-6 is primarily controlled by *cnd-1*, with a weak contribution from *ceh-13*. However, *unc-25p*::*GFP* expression in DD1 and DD2 is redundantly controlled by both *cnd-1* and *ceh-13*. An alternative interpretation is that both *cnd-1* and *ceh-13* are required for successful induction of ABpl/rppapp fates. Loss of these genes may mean that this cell division is lost, leading to a default anterior fate that permits aspects of DA2-6 to be specified normally but leads to loss of all DD neurons. (E) model summarizing *cnd-1*, *ceh-5*, and *ceh-13* function in the control of RME sub-module transcription and DD neuron fate specification.

Our comparative transcriptome data expand on *cnd-1*’s role as a proneural transcription factor, identifying the homeobox gene *ceh-5* as a novel downstream target of *cnd-1* (Figure 1). We find that *cnd-1* controls *ceh-5* gene expression in the RME neurons, suggesting that *ceh-5* may function as a terminal selector transcription factor in this cell type. Analysis of publicly available single-cell RNA-seq data allows us to contextualize the relationship between *cnd-1* and *ceh-5* (Packer *et al.* 2019). Figure 7 shows sub-lineages of single-cell RNA-seq data visualized using the Viscello package. Onset of *cnd-1* expression in the RME parent cell lineages (Figure 7A) occurs at the same time as onset of *ceh-5* expression (Figure 7B). However, our *ceh-5p*::*GFP* reporter gene data suggests that *cnd-1* controls *ceh-5* expression in the RME neurons. It may be that the single-cell RNA-seq data lacks the temporal resolution to define when one transcription factor is transcribed relative to another. Alternatively, *cnd-1* and *ceh-5* may function collaboratively to maintain *ceh-5* expression, for instance in other head neuron and muscle cell types, where we see *ceh-5p*::*GFP* expression drop but is not eliminated in *cnd-1* mutants. It should be noted that some aspects of RME neuron fate appear to be preserved in *cnd-1* mutants as they continue to express *unc-25p*::*GFP* (a known RME marker gene), even when *unc-25p*::*GFP* is lost in posterior DD neurons (Figure 5 and 6). This suggests that *ceh-5* may control a sub-module of RME terminal fate but not the actual fate of the neuron itself. The co-expression of *cnd-1* and *ceh-13* in terminal fate cells such as the RMEs may be predictive of *cnd-1*’s ability to control *ceh-5* expression in other neurons. Viscello data shows that these two transcription factors are co-expressed in RIVL/R, FLPL/R and PVQL/R neurons (Packer *et al.* 2019). Our data shows that *cnd-1**(**gk718**)* mutants lose *ceh-5p*::*GFP* expression in PVQ neurons, supporting this hypothesis. Overall, our data adds to previously published work, placing *cnd-1* as a proneural transcription factor upstream of *ceh-5*, *unc-3*, *unc-4*, and *unc-47*, to control aspects of RME, PVQ, DA, DB, and DD neuron fate respectively (Miller *et al.* 1992; Jin *et al.* 1994; Prasad *et al.* 1998; Kratsios *et al.* 2011).

### CND-1 regulation of cnd-1-expressing cells

Our RNA-seq data shows that *cnd-1* transcription appears to be up-regulated in *cnd-1**(**ju29**)* animals (Table 1B, Figure 2B and C). While there is evidence of intron inclusion, it does not appear to be sufficient to explain the almost twofold increase in *cnd-1**(**ju29**)* transcript levels. We postulate that the intron inclusion may positively affect transcript stability, leading to higher levels of transcript for longer. We used two separate reporter gene assays to further explore the role of *cnd-1* in nervous system development. First, quantitative imaging of *cnd-1p*::*his24*::*mCherry* in comma-stage embryos shows reduced *cnd-1* expression in the two *cnd-1* mutant alleles examined. Second, *cnd-1p*::*his24*::*mCherry* and *unc-25p*::*GFP* assays in ventral cord motorneurons reveals significantly lower cell counts in *cnd-1* mutants, with *cnd-1**(**ju29**)* mutants displaying strong DD neuron induction defects, although not as strong as those seen in *cnd-1**(**gk718**)* mutants. This indicates that *cnd-1* is required for the fate specification of cells that normally express *cnd-1*. Whether this is via a self-regulatory mechanism is not known.

### CND-1 functions redundantly with CEH-13 to specify DD1 and DD2 cell fate

Our data corroborate previous work showing that loss-of-function in the Hox gene *ceh-13*/labial leads to loss of ventral cord motorneurons in a manner similar to that seen in *cnd-1* mutants (Hallam *et al.* 2000; Stefanakis *et al.* 2015). While *ceh-13* is not significantly different in our whole embryo RNA-seq dataset, a previous RNA-seq study, using Fluorescence-Activated Cell Sorting to enrich for *cnd-1*-labeled embryonic cells, revealed that *ceh-13* transcripts are up-regulated in that tissue relative to background (Burdick *et al.* 2016). This led us to investigate the genetic interaction between these two highly conserved transcription factors with regard to DD neuron fate specification (Figures 5 and 6). While both genes show similar loss-of-function DD neuron phenotypes, there are subtle differences. In *cnd-1**(**gk718**)* mutants, cell fate specification of DD1 and DD2 ventral motorneurons appears normal, whereas DD3-6 are generally eliminated (Figure 5B and C). However, *ceh-13**(**sw1**)* mutants show a weaker, variable loss of DD3-6, but with robust induction of DD1 and perhaps DD2. Analysis of *cnd-1**(**gk718**) **ceh-13**(**sw1**)* double mutants reveals a striking synergy with almost complete loss of all DD neurons (Figure 5F and G). This is not due to a change of cell fate, as analysis of all ventral nerve cord cells shows a corresponding cell count difference that mirrors the loss of DD neurons (Figure 6). Based on total ventral cord cell counts, we tentatively conclude that DA and DB motorneurons are not obviously affected by loss of *cnd-1* and/or *ceh-13*. This is in contrast to previously published work showing less cholinergic DA and DB ventral cord neurons when labeled by *acr-2p*::*YFP* (Hallam *et al.* 2000). One possible explanation for this discrepancy may be the transgenes used to label the cells. Hallam *et al.* (2000) used *acr-2p*::*YFP* and *unc-25p*::*GFP* (which are driven from a cholinergic receptor and glutamate decarboxylase promoter sequences respectively) to label all embryonic motorneurons and reported that some *cnd-1**(**ju29**)* ventral cord cells lacked expression of both reporters. Despite this, those cells were apparently present, based on the stereotyped location of cell bodies and nuclei identified using Differential Interference Contrast microscopy. The reporter gene used in our assay was driven from a *rab-3* promoter element that was previously shown to label all neurons apart from the CAN associated neurons (Stefanakis *et al.* 2015). It may be that all DA and DB type neurons are born in *cnd-1* and/or *ceh-13* mutants but lack a complete battery of terminal selectors leading to apparent loss of identity, depending on the reporter gene used to label that cell.

Figure 7C and D shows the relationship between *cnd-1* and *ceh-13* transcript expression as identified via single-cell RNA-seq (Packer *et al.* 2019). Similar to the relationship between *cnd-1* and *ceh-5*, there does not appear to be any temporal sequence in their expression. Intriguingly, this visualization shows loss of *cnd-1* and *ceh-13* expression in the DD1 and DD2 mother cells (ABplppappa and ABprppappa respectively). We speculate that this renders DD1 and DD2 resistant to changes in either *cnd-1* or *ceh-13* expression, such that both cells are correctly specified in either single mutant background. Perhaps loss of both *cnd-1* and *ceh-13* promotes premature cell cycle exit in DD mother cells (as postulated by Hallam *et al.* 2000), preventing any DD neurons (and presumably RIGL and RIGR) from being born. While the DA2-5 mother cells express *cnd-1* and *ceh-13*, their grandparents (ABplppapa and ABprppapa) only express *cnd-1* (and at a lower level than the posterior daughter). This raises the possibility that this anterior branch of the lineage is less sensitive to these transcription factors and may give rise to a default set of cell fates, which means that DA2-5 are born whereas DD1-6 are lost. Figure 7E summarizes our analysis on the genetic interactions between *cnd-1* and *ceh-13* and the sub-fate terminal selector transcription factors *ceh-5* in RME and *unc-30* in DD neurons respectively. *unc-30* was previously reported to control the GABAergic neurotransmission module of DD and RME neurons (Eastman *et al.* 1999). While our RNA-seq assay does not reveal significant changes in *unc-30* expression, it may have been below the threshold for statistical significance.
